# Inhibition of metastasis, angiogenesis, and tumor growth by Chinese herbal cocktail Tien-Hsien Liquid

**DOI:** 10.1186/1471-2407-10-175

**Published:** 2010-04-30

**Authors:** Jean-San Chia, Jia-Ling Du, Wei-Bin Hsu, Andy Sun, Chun-Pin Chiang, Won-Bo Wang

**Affiliations:** 1 Graduate Institute of Microbiology, National Taiwan University, Taipei 100, Taiwan; 2 School of Dentistry, College of Medicine, National Taiwan University, Taipei 100, Taiwan

## Abstract

**Background:**

Advanced cancer is a multifactorial disease that demands treatments targeting multiple cellular pathways. Chinese herbal cocktail which contains various phytochemicals may target multiple dys-regulated pathways in cancer cells and thus may provide an alternative/complementary way to treat cancers. Previously we reported that the Chinese herbal cocktail Tien-Hsien Liguid (THL) can specifically induce apoptosis in various cancer cells and have immuno-modulating activity. In this study, we further evaluated the anti-metastatic, anti-angiogenic and anti-tumor activities of THL with a series of *in vitro *and *in vivo *experiments.

**Methods:**

The migration and invasion of cancer cells and endothelial cells was determined by Boyden chamber transwell assays. The effect of THL on pulmonary metastasis was done by injecting CT-26 colon cancer cells intravenously to syngenic mice. The *in vitro *and *in vivo *microvessel formation was determined by the tube formation assay and the Matrigel plug assay, respectively. The *in vivo *anti-tumor effect of THL was determined by a human MDA-MB-231 breast cancer xenograft model. The expression of metalloproteinase (MMP)-2, MMP-9, and urokinase plasminogen activator (uPA) was measured by gelatin zymography. The expression of HIF-1α and the phosphorylation of ERK1/2 were determined by Western blot.

**Results:**

THL inhibited the migration and invasion ability of various cancer cells *in vitro*, decreased the secretion of MMP-2, MMP-9, and uPA and the activity of ERK1/2 in cancer cells, and suppressed pulmonary metastasis of CT-26 cancer cells in syngenic mice. Moreover, THL inhibited the migration, invasion, and tube formation of endothelial cells *in vitro*, decreased the secretion of MMP-2 and uPA in endothelial cells, and suppressed neovascularization in Matrigel plugs in mice. Besides its inhibitory effect on endothelial cells, THL inhibited hypoxia-induced HIF-1α and vascular endothelial growth factor-A expression in cancer cells. Finally, our results show that THL inhibited the growth of human MDA-MB-231 breast cancer xenografts in *NOD-SCID *mice. This suppression of tumor growth was associated with decreased microvessel formation and increased apoptosis caused by THL.

**Conclusion:**

Our data demonstrate that THL had broad-spectra anti-cancer activities and merits further evaluation for its use in cancer therapy.

## Background

Metastasis, or the spread of cancer from its primary site to a distant organ, is the main cause of death in patients with malignancy [1]. Metastasis of cancer cells involves multiple processes and various cytophysiological changes [2]. To metastasize, cancer cells first lose the ability to adhere to neighboring tumor cells and gain migratory and invasive capabilities. Cancer cells can then permeate the basement membrane, invade surrounding tissues and gain direct access to blood and lymphatic vessels via which cancer cells can disseminate throughout the body. During this process, degradation of the extracellular matrix and components of the basement membrane by proteases, such as matrix metalloproteinase (MMP)-2, MMP-9, and urokinase plasminogen activator (uPA), plays a critical role in tumor invasion and metastasis [3-6]. Patients with metastatic cancers can no longer be cured by local therapy alone and usually die after painful chemotherapy. Thus control of cancer metastasis is an important issue in tumor treatment.

Angiogenesis, the process of new blood vessel formation, plays a crucial role in the growth and metastasis of tumors [7]. Tumor growth and progression require angiogenesis because in its absence tumor growth is restricted to a few millimeters in diameter due to the physical constraint set by simple diffusion of nutrients and oxygen. In addition, angiogenesis and vascularization allows metastatic tumor cells escape into the circulation and lodge in other organs [7,8]. As a tumor expands, local hypoxic conditions induce a molecular response in tumor cells, leading to the activation of a key transcription factor, the hypoxia-inducible factor (HIF) [9]. This transcription factor induces the expression of pro-angiogenic growth factors, such as vascular endothelial growth factor (VEGF), which in turn bind to and activate their respective receptors on the surface of endothelial cells, leading to angiogenesis [10,11]. Since angiogenesis plays a prominent role in tumor growth and metastasis, inhibition of angiogenesis is considered to be an important strategy for cancer therapy [12,13].

Advanced cancer is a multifactorial disease that demands treatments targeting multiple cellular pathways. The fact that chemotherapy using cytotoxic anti-cancer drugs has significant side effects and offers little survival benefit for patients with advanced malignancies has prompted the use of alternative medicine in cancer treatment. Chinese/Oriental herbal medicine, an ancient and complete medicinal system based on empirical observations, has long been used for treatment of malignancies. Whereas single herbs are seldom used alone for cancer treatment, herbal cocktails containing extracts from several herbs are often used. A number of herbal cocktails have been reported to have anti-tumor activities [14-19] and some of them have been used by cancer patients for many years. However, herbal remedies are yet to be integrated into main stream medicine mainly due to lack of experimental and clinical studies on their safety, efficacy, and pharmacological mechanisms [20]. Careful *in vitro *and *in vivo *studies will be essential and necessary to evaluate their efficacy and safety before clinical trials can be contemplated.

Herbal cocktail may target multiple cellular pathways to correct the dys-regulated cellular functions accompanying different stages of cancer development. It is believed that a properly formulated herbal cocktail which takes advantage of synergy and interactions among a myriad of phytochemicals present in the different herbs may achieve better therapeutic efficacy than single herbs. The Chinese herbal cocktail, Tien-Hsien Liquid (THL, prepared by China-Japan Feida Union Co., Ltd., Hong Kong), is a herbal mixture that has been used as an anticancer dietary supplement for more than 15 years and has been used by many cancer patients with favorable results in over 15 countries. Moreover, our previous studies indicate that it has immuno-modulating activity [21,22] and can specifically induce apoptosis in a wide variety of cancer cells [23]. THL is an aqueous preparation of herbal mixture and consists mainly of extracts from 14 Chinese medicinal herbs: *Cordyceps sinensis *(CS), *Oldenlandia diffusa *(OD), *Indigo pulverata levis *(IPL; also known as *Indigo Naturalis*), *Polyporus umbellatus *(PU), *Radix astragali *(RA), *Panax ginseng *(PG), *Solanum nigrum L*. (SNL), *Pogostemon cablin *(PC), *Atractylodis macrocephalae rhizoma *(AMR), *Trichosanthes radix *(TR), *Clematis radix *(CR), *Margarite *(M), *Ligustrum lucidum Ait *(LLA), and *Glycyrrhiza radix *(GR) [21-23]. Among these constituent herbs, the following herbs or their components have been shown to have anti-tumor activity: CS [24-27], OD [28,29], IPL [30,31], PU [32,33], RA [34-37], PG [38-40], SNL [41], TR [42,43], CR [44], LLA [34], and GR [45,46]. Moreover, whereas CS [27,47], PG [48,49], GR [50,51] and OD [52] have been shown to inhibit tumor metastasis, PG [39,48,49], GR [53] and SNL [54] have been shown to have anti-angiogenic activity. These results suggest that THL may have inhibitory effect on tumor growth, metastasis and angiogenesis. In this study, we evaluated the anti-metastatic, anti-angiogenic and anti-tumor effects of THL with a series of *in vitro *and *in vivo *pre-clinical experiments. Our data indicate that THL had anti-metastatic, anti-angiogenic, and anti-tumor activities. These results support the merit of this herbal cocktail for therapy of various cancers.

## Methods

### Cell culture

The human lung carcinoma cell line, H1299, and the mouse colon carcinoma cell line, CT-26, were routinely grown in Dulbecco's modified Eagle medium (GIBCO BRL Life Technologies, Grand Island, NY) supplemented with 10% fetal bovine serum (FBS) in 5% CO_2_. The human breast adenocarcinoma cell line, MDA-MB-231, was cultured in DMEM/F-12 1:1 medium (GIBCO BRL Life Technologies) supplemented with 10% FBS in 5% CO_2_. The human prostate adenocarcinoma cell line, PC-3, was cultured in RPMI-1640 medium (GIBCO BRL Life Technologies) supplemented with 10% FBS in 5% CO_2_. The human microvascular endothelial cell line-1 (HMEC-1) was cultured in MCDB131 medium (GIBCO BRL Life Technologies) supplemented with 10% FBS, 10 ng/ml epidermal growth factor (Becton Dickinson, San Jose, CA), and 1 mg/ml hydrocortisone (Sigma-Aldrich, Inc., St. Louis, MO) in 5% CO_2_. Primary human umbilical vein endothelial cells (HUVEC) were isolated from umbilical cord as described [55] and maintained in medium 199 (GIBCO BRL Life Technologies) supplemented with 20% FBS, 30 μg/ml endothelial cell growth supplement (Upstate Biotechnology, Lake Placid, NY), 15 μg/ml heparin (Leo Pharmaceutical Product, Ballerup, Denmark), and 1 mM pyruvate in 5% CO_2_.

### Handling of THL

For the *in vitro *experiments, the culture medium containing THL was prepared as follows. THL (obtained from China-Japan Feida Union Co., Ltd., Hong Kong) was centrifuged to remove insoluble ingredients, and the supernatant was added to the appropriate culture medium to the final concentrations of 0.1, 0.25, 0.5, 0.75 or 1% (v/v). For the *in vivo *pulmonary metastasis experiment, THL was used directly (without centrifugation before use). For the breast cancer xenograft experiment, THL was centrifuged to remove insoluble ingredients, and the supernatant was diluted with phosphate-buffered saline (PBS) at 1:1 (v/v) ratio.

### Preparation of conditioned medium of MDA-MB-231 cancer cells (231-CM)

2 × 10^6 ^MDA-MB-231 cells were incubated for 24 h in 5 ml of serum-free medium. The medium was then collected, filtrated to remove cell debris, and stored at -20°C until use. For mouse Matrigel plug assays, the conditioned medium was concentrated 50 fold before use.

### Wound healing migration assay

Cancer cells were seeded at a density of 1-5 × 10^5^cells/well in 12-well culture plates and allowed to form a confluent monolayer. The layer of cells was then scraped with a 20-200 μl micropipette tip to create a wound of ~1 mm width. Cells were then washed twice with fresh medium, and replaced with medium containing indicated concentration of THL. After incubation at 37°C for 20 h, cells were washed with PBS, fixed with 4% paraformaldehyde, and stained with 0.5% Coomassie Brilliant Blue. Images of the wounds were captures at 0 h and 20 h after scraping at 100-fold magnification and the average distance of the wound was calculated by using Image Pro Plus software (Media Cybernetics, Bethesda, MD). The ability of the cells to close the wound, that is, their motility, was calculated in the following way: (average distance of the wound at 0 h - average distance of the wound at 20 h/average distance of the wound at 0 h) × 100.

### Migration and invasion assays

The *in vitro *cell migration and invasion assays were performed by using a modified Boyden chamber inserted with polyethylene terephthalate filter membrane containing 8-μm pores in 24-well plates (Millipore, Billerica, MA). For cell invasion assays, the filter membranes were coated with Matrigel (30 μg, Becton Dickinson, San Jose, CA).

Cells (1 × 10^5^) suspended in 200 μl of serum-free medium were seeded onto the upper compartment of the transwell chamber. The lower chamber was filled with serum-free medium containing chemoattractants (10% FBS for migration and invasion of cancer cells; 231-CM for migration and invasion of endothelial cells) and various concentrations of THL. After incubation for 6 h (for migration assays) or 24 h (for invasion assays), the medium in the upper chamber was removed and the filters were fixed with 70% ethanol for 10 min. The cells remaining on the upper surface of the filter membrane were then completely removed by wiping with a cotton swab, and the cells on the opposite surface of the filter membrane were stained with 0.5% Coomassie Brilliant Blue for 10 min. The migrated/invaded cells were then visualized and counted from six randomly selected fields (× 200 magnification) under an inverted microscope.

### Zymography

Production of MMPs and uPA by cancer or endothelial cells were analyzed by gelatin and plasminogen-casein zymography, respectively. In MMP gelatin zymography, cells were cultured in serum-free medium with various concentration of THL for 24 h (cancer cells) or 6 h (endothelial cells), and conditioned media were collected, filtrated, and concentrated (~50 fold). Equal amount of conditioned medium samples were mixed with SDS sample buffer containing 2% SDS without β-mercaptoethanol and applied, without boiling, to 7.5% SDS polyacrylamide gels copolymerized with 2 mg/ml gelatin (Sigma-Aldrich, Inc., St. Louis, MO). After electrophoresis, gels were washed for 30 min at room temperature with gentle agitation in renaturing buffer (2.5% Triton X-100 in H_2_O) to remove SDS. The gels were then equilibrated in developing buffer (40 mM Tris-HCl, pH 7.4, 200 mM NaCl, 10 mM CaCl_2_) at room temperature with gentle agitation for 30 min. After removing the old developing buffer, the gels were incubated in fresh developing buffer at 37°C overnight. The gels were then stained with 0.5% Coomassie Brilliant Blue and destained. The MMP activities were visualized as clear bands against the blue background of the stained gels. The uPA zymography was performed as described in the MMP gelatin zymography, except that the SDS polyacrylamide gels containing 1 mg/ml casein (MP Biomedicals, Inc., Irvine, CA) and 1 U/ml plasminogen (MP Biomedicals, Inc.) were used.

### Pulmonary metastasis assay

All animal experiments in this study were performed following the Guidelines for Animal Experiments in National Taiwan University and were approved by the Institutional Animal Care and Use Committee in College of Medicine, National Taiwan University (IACUC Approval No: 20060184). Balb/c female mice (6-8 weeks old) were purchased from Laboratory Animal Center at College of Medicine, National Taiwan University (Taipei, Taiwan) and given food and water *ad libitum*. The mice were oral fed with either THL or water (200 μl; twice a day) throughout the experimental duration. On Day 8 of treatment, the mice were injected intravenously (via tail veins) with 2 × 10^5 ^mouse CT-26 colon cancer cells to establish pulmonary metastasis. Mice were killed 15 days after tumor cell injection and the metastatic nodules on the surface of the lungs were counted. The lungs were fixed with formalin. Thin sections were stained with hematoxylin and eosin. Representative fields (at × 40 or × 100 magnification) for each group were photographed.

### Tube formation assay

Tube formation of HUVEC and HMEC-1 endothelial cells on Matrigel was performed as described [56]. Matrigel (200 μl) was added to 24-well plates and allowed to solidify for 30 min at 37°C. Endothelial cells (5 × 10^4 ^cells/well) suspended in 500 μl of complete medium or 231-CM containing various concentration of THL were seeded on the solidified Matrigel. After incubation for 5 h, cells were fixed with 4% paraformaldehyde and stained with 0.1% crystal violet in 20% methanol. Randomly chosen fields were photographed at × 100 magnification and the closed networks of vessel-like tubes were counted.

### Mouse Matrigel plug assay

Female *NOD-SCID *mice (6-8 weeks old) were purchased from Laboratory Animal Center at College of Medicine, National Taiwan University and housed in pathogen-free condition. The mice were subcutaneously injected with 500 μl of Matrigel containing concentrated 231-CM (10 μl), heparin (10 U) and either THL (5 μl) or water (5 μl). Fourteen days later, mice were killed and the Matrigel plugs were removed. To quantitate the formation of functional blood vessel, the amount of hemoglobin was measured using the Drakin's reagent kit (Sigma-Aldrich, St Louis, MO).

### Human MDA-MB-231 breast cancer xenograft model

Female *NOD-SCID *mice (6-8 weeks old) were purchased from Laboratory Animal Center at College of Medicine, National Taiwan University and housed in pathogen-free condition throughout the experimental duration. Mice were given free access to commercial rodent chow and water. MDA-MB-231 cancer cells (3 × 10^6^, suspended in 100 μl of PBS) were injected subcutaneously into both flanks of the mouse. One week after tumor cell inoculation, the mice were randomly divided into two groups. The weight of the mice in these two groups was similar at this time point. One group was intraperitoneally injected with 100 μl of PBS-diluted THL [1:1 (v/v) dilution] once a day until the end of the experiment (It should be noted that the dosage of THL was optimized in our preliminary experiments and that the THL dose used here showed no toxic effect to the mice). The other group was administrated with PBS using similar protocol as described above. The mouse body weight and tumor size were measured at different time points following tumor implantation, and the tumor volume was calculated according to the following formula: 1/2 (Length × Width^2^). The tumors were removed at Day 36 after tumor implantation, photographed, and weighed. The tumors were snap-frozen in liquid nitrogen for immunohistochemical (IHC) and TUNEL analyses.

### IHC and TUNEL analyses

Cryostat sections of frozen tumors were fixed with 4% paraformaldehyde, washed with PBS, and the endogenous peroxidase activity was blocked with Dako Dual endogenous enzyme block (Dako, Glostrup, Denmark). After washing with PBS, the sections were blocked with 5% FBS in PBS. To detect CD31-positive stained microvessels, the sections were probed with rat anti-CD31 antibody **(**Becton Dickinson, San Jose, CA), and then incubated with horseradish peroxidase-conjugated secondary antibody by using Rat on Mouse HRP-Polymer Kit (Biocare Medical, Concord, CA). Following color development by using Dako DAB reagent (Dako), the nuclei were stained with hematoxyline. The sections were then sealed with glycerol-gelatin (Sigma-Aldrich, Inc., St. Louis, MO) for microscopic observation. Randomly chosen fields were photographed at × 200 magnification and the number of CD31-positive stained blood vessels was counted. For TUNEL assay, the cryostat sections were fixed in 4% paraformaldehyde, washed with PBS and permeated with permeabilization solution (0.1% Triton X-100, 0.1% sodium citrate in PBS). The sections were then labeled with TUNEL reaction mixture according to the protocol provided by the manufacturer (Roche Applied Science, Mannheim, Germany) to detect apoptotic cells. Following TUNEL reaction, the sections were rinsed three times with PBS, and incubated in Hoechest 33258 solution to label nuclear DNA. The sections were then sealed with mounting medium (Sigma-Aldrich, Inc.) and subjected to fluorescence microscopy. Randomly chosen fields were photographed at × 200 magnification and the number of TUNEL-positive cells was counted.

### Statistical analyses

Data are present as the mean ± SD. The significance of the difference between groups was evaluated with the Student's t-test, p < 0.05 was considered significant.

## Results

### THL inhibits the migration ability of various cancer cells

**Figure 1:**
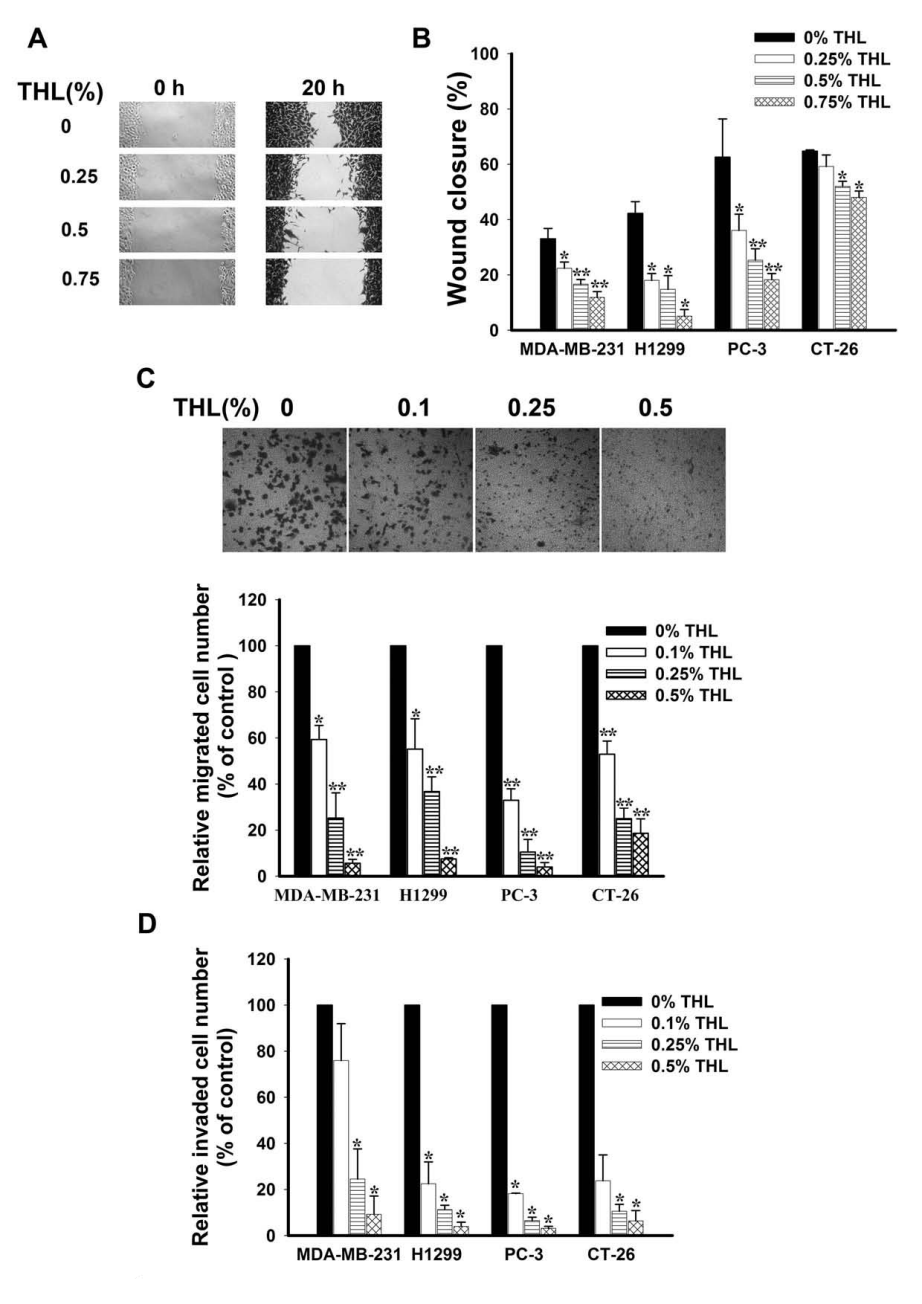
**THL inhibits the migration and invasion of MDA-MB-231, H1299, PC-3, and CT-26 cancer cells**. A) Images of wound healing assays (× 100 magnification). B) Wound healing assays indicate that THL inhibited the migration of various cancer cells dose-dependently. Values represent means ± SD, n = 3. *P < 0.05; **P < 0.001 versus untreated control. C) Boyden chamber transwell assays indicate that THL dose-dependently inhibited the migration of various cancer cells. *Upper panel*, images of migrated MDA-MB-231 cancer cells (× 200 magnification). *Lower panel*, quantitation of migrated cancer cells. The migrated cells were counted in six randomly selected microscopic fields (× 200 magnification). The relative migrated cell number was obtained by comparing the migrated cell number in the presence of THL with that in the absence of THL. Values represent means ± SD, n = 3. *P < 0.05; **P < 0.001 versus untreated control. D) THL dose-dependently inhibited the invasion of various cancer cells. The cells invaded to the lower chamber were counted in six randomly selected microscopic fields (× 200 magnification). The relative invaded cell number was obtained by comparing the invaded cell number in the presence of THL with that in the absence of THL. Value represent means ± SD, n = 2. *P < 0.05 versus untreated control.

Metastasis consists of sequential steps involving cancer cell migration and invasion. To study whether THL had an anti-metastatic effect, four highly invasive cancer cell lines, including PC-3 (human prostate cancer cells), MDA-MB-231 (human breast cancer cells), H1299 (human lung cancer cells), and CT-26 (mouse colon cancer cells) [57-60], were used. We first tested the effect of THL on the motility of cancer cells by the wound healing assay. Confluent PC-3 cancer cells were scraped with a sterilized tip as shown in Fig 1A, left panel. Cancer cells were then allowed to migrate into the gap created by the scraping either in the absence or presence of THL. After 20 h incubation, the gap remained unfilled by the migrated cells in the THL-treated group was wider than that in the untreated group (Fig 1A, right panel), indicating that THL can inhibit the motility of PC-3 cancer cells. Similar wound healing assays were also performed with MDA-MB-231, H1299, and CT-26 cancer cells in the presence of various concentrations of THL. As shown in Fig 1B, THL could inhibit the migration ability of all these cancer cells in a dose-dependent manner. We also tested the effect of THL on the migration of MDA-MB-231, H1299, PC-3, and CT-26 cancer cells by the Boyden chamber transwell assay. In all the cell lines tested, the number of cells migrated to the lower chamber was reduced by THL in a concentration-dependent manner (Fig 1C). The inhibitory effect of THL on the migration of these cancer cells is not due to the cytotoxic effect of THL, because the viability of these cancer cells was barely affected by THL in the concentration range tested (see Additional file 1: Supplemental figure S1). Together, these data further confirm that THL can inhibit the migration ability of cancer cells.

### THL inhibits the invasion ability of cancer cells

We next tested whether THL could inhibit the invasion ability of cancer cells. The invasion property of MDA-MB-231, H1299, PC-3, and CT-26 cancer cells was analyzed in the Matrigel-coated Boyden chamber in the presence of various concentrations of THL. In all the cell lines tested, the number of cells invaded through the Matrigel-coated filter was reduced by THL dose-dependently (Fig 1D), indicating that THL can inhibit the invasion ability of these cancer cells.

**Figure 2:**
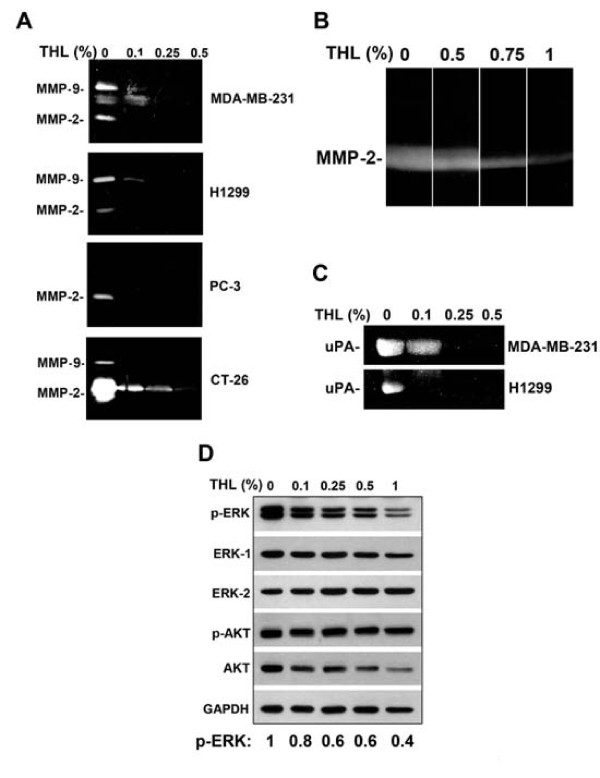
**THL inhibits the secretion of MMP-2, MMP-9 and uPA and the activity of ERK1/2 in cancer cells**. A) Gelatin zymogram of concentrated conditioned medium from MDA-MB-231, H1299, PC-3, or CT-26 cancer cells treated with various concentrations of THL during 24-h incubation period. B) Gelatin zymogram showing direct inhibitory effect of THL on MMP-2 activity. C) Casein zymogram of concentrated conditioned medium from MDA-MB-231 or H1299 cancer cells treated with various concentrations of THL during 24-h incubation period. D) Western blot analysis shows that THL inhibits the phosphorylation of ERK1/2 but not that of AKT in CT-26 cancer cells. CT-26 cells were treated with various concentrations of THL in serum-free medium for 24 h. Western blot analysis of cell lysates was performed as described previously [66]. The blot was first probed with antibodies against phospho-ERK1/2 (cell signaling, Danvers, MA) and phospho-AKT (cell signaling), and then reprobed with antibodies against ERK1, ERK2 (Santa cruz, Santa Cruz, CA) and AKT (cell signaling). Glyceraldehyde-3-phosphate-dehydrogenase (GAPDH) served as an internal control for amounts of protein loaded on the gel.

MMP-2, MMP-9, and uPA are known to be involved in the degradation of extracellular matrix and play a critical role in tumor invasion and metastasis. We thus tested whether THL could inhibit the secretion of MMP-2, MMP-9, and uPA in cancer cells. Gelatin zymography assays indicated that THL dose-dependently inhibited the secretion of MMP-2 and MMP-9 in MDA-MB-231, H1299, PC-3 and CT-26 cancer cells (Fig 2A). Real-time RT-PCR analysis indicated that THL could inhibit the transcription of the MMP-2 and MMP-9 genes in MDA-MB-231 cells (see Additional file 2: Supplemental figure S2). We also examined whether THL directly affected MMP enzymatic activity. Conditioned medium of untreated CT-26 cells was loaded into a single wide lane on gelatin-containing gel, electrophoresed, and the gel was divided into four strips. The gel strips were then incubated in developing buffer containing various concentrations of THL. As shown in Fig 2B, THL could inhibit MMP-2 activity directly in a dose-dependent manner. To study whether THL could inhibit the secretion of uPA in cancer cells, casein zymography assays were performed. As shown in Fig. 2C, THL dose-dependently inhibited the secretion of uPA in MDA-MB-231 and H1299 cells.

The extracellular signal-regulated kinase (ERK) signaling pathway is known to up-regulate the expression of MMPs [61]. We thus tested the effect of THL on the ERK signaling pathway. We found that THL could inhibit the phosphorylation of ERK1/2 but not that of AKT (Fig 2D) in CT-26 cancer cells, indicating that THL can inhibit the ERK signaling pathway but not the phosphatidylinositol-3-OH kinase (PI3K)/AKT signaling pathway.

### THL inhibits the pulmonary metastasis of mouse CT-26 colon cancer cells in mice

**Figure 3:**
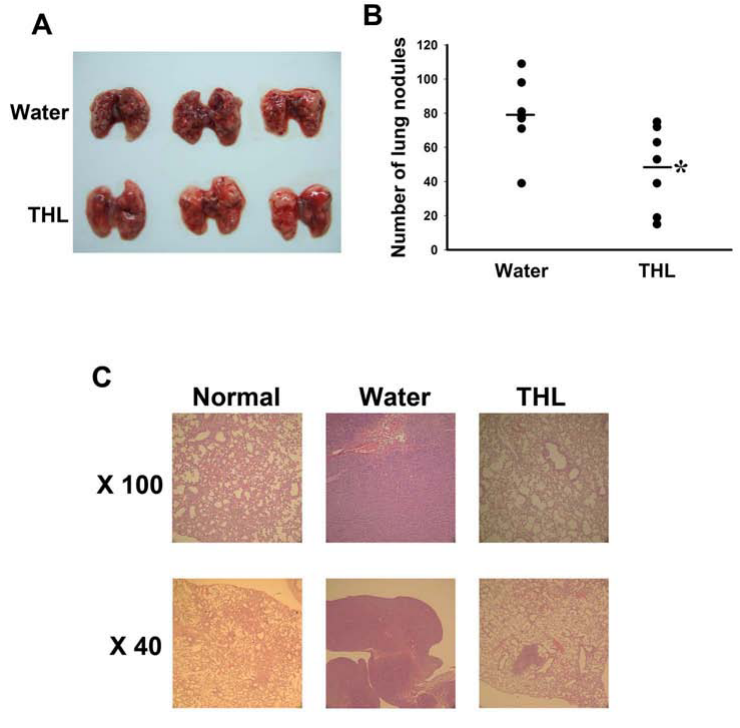
**THL inhibits the pulmonary metastasis of mouse CT-26 cancer cells in Balb/c mice**. A) Images of metastatic lung nodules following water or THL treatment. B) Quantitation of metastatic lung nodules. *P < 0.05 versus water-treated mice. C) Histological appearance of representative lungs from normal (not injected with tumor cells), water-treated, and THL-treated mice.

In light of the above findings, we next tested the ability of THL to inhibit the experimental lung metastasis produced by an intravenous injection of CT-26 cancer cells to the syngenic Balb/c mice. The mice were fed THL or water throughout the experimental duration. Fifteen days after tumor inoculation, the mice were sacrificed and the tumor nodules on the surface of the lung were counted and photographed (Fig 3A). The average number of tumor nodules in water-fed group was 79 while that in the THL-fed group was 48 (Fig 3B), indicating that THL treatment significantly decreased tumor colonization in the lung. Histological examination of the lung sections showed that the lungs of the water-fed mice were filled with metastasized CT-26 cells (Fig 3C). In contrast, the lungs of the THL-fed mice contained much less metastasized CT-26 cells and looked much more normal morphologically (Fig 3C). Together, these results strongly suggest that THL can inhibit cancer metastasis and could be useful for prevention of cancer metastasis and recurrence in cancer patients.

### THL inhibits the migration and invasion ability of endothelial cells

**Figure 4:**
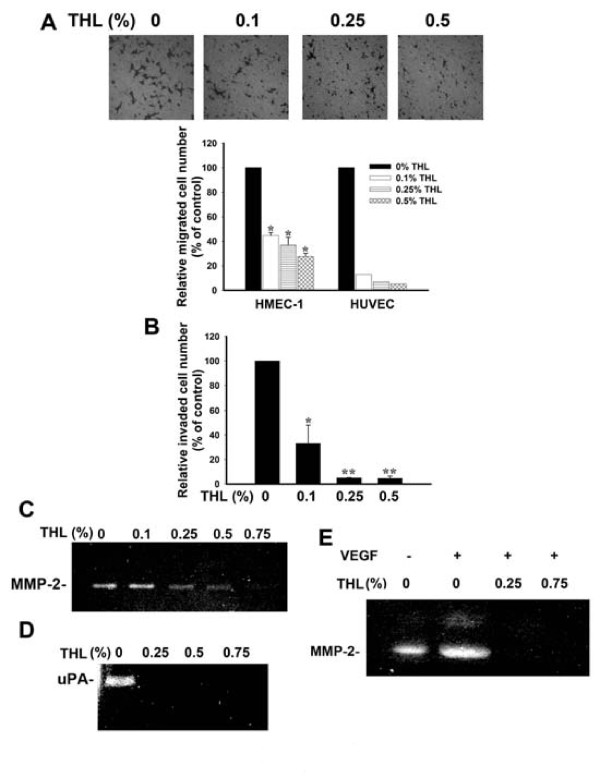
**THL inhibits the migration and invasion ability of endothelial cells and inhibits the secretion of MMP-2 and uPA in endothelial cells**. A) The Boyden chamber transwell assay indicates that THL can dose-dependently inhibit the migration of HMEC-1 and HUVEC endothelial cells. *Upper panel*, images of migrated HMEC-1 endothelial cells in the presence of various concentrations of THL (× 200 magnification). *Lower panel*, quantitation of migrated HMEC-1 and HUVEC endothelial cells in the presence of various concentrations of THL at 6 h post-seeding. The migrated cells were counted in six randomly selected microscopic fields (× 200 magnification). The relative migrated cell number was obtained by comparing the migrated cell number in the presence of THL with that in the absence of THL. Values represent means ± SD, n = 2. *P < 0.05 versus untreated control. B) The Matrigel-coated Boyden chamber transwell assay indicates that THL can dose-dependently inhibit the invasion ability of HMEC-1 endothelial cells. The cells that invaded and migrated to the lower chamber were counted in six randomly selected microscopic fields (× 200 magnification). The relative invaded cell number was obtained by comparing the invaded cell number in the presence of THL with that in the absence of THL. Values represent means ± SD, n = 2. *P < 0.05; **P < 0.001 versus untreated control. C) Gelatin zymogram of concentrated conditioned medium from HMEC-1 endothelial cells treated with various concentrations of THL during 6-h incubation period. D) Casein zymogram of concentrated conditioned medium from HMEC-1 endothelial cells treated with various concentrations of THL during 6-h incubation period. E) Gelatin zymography indicates that THL can inhibit VEGF-induced MMP-2 expression in HMEC-1 endothelial cells. The cells were cultured in serum-free medium without or with VEGF (50 ng/ml) plus various concentrations of THL for 6 h.

Angiogenesis plays an important role in tumor growth and metastasis. Angiogenesis consists of multiple steps including endothelial cell proliferation, migration, invasion and tube formation. To test whether THL could inhibit angiogenesis, we first tested the effect of THL on endothelial cell migration by Boyden chamber transwell assay. Endothelial cells (HMEC-1 or HUVEC) plated on the upper chamber containing serum-free medium were allowed to migrate to the lower chamber containing MDA-MB-231 breast cancer cell-derived conditioned medium (231-CM, serves as a chemo-attractant) plus various concentrations of THL. As shown in Fig 4A, the migration of HMEC-1 and HUVEC toward 231-CM was suppressed by THL in a dose-dependent manner. This inhibition of migration is not due to the cytotoxic effect of THL, because the viability of these cells was not affected by THL in the concentration range tested (see Additional file 3: Supplemental figure S3). Together, these data suggest that THL can inhibit the migration of endothelial cells to cancer cells. We also investigated the effect of THL on HMEC-1 invasion by using 231-CM as chemo-attractant in a Matrigel-coated Boyden chamber assay. As shown in Fig 4B, the number of HMEC-1 cells invaded through the Matrigel-coated filter was reduced by THL dose-dependently, indicating that THL can inhibit the invasion ability of endothelial cells.

MMP-2 and uPA are known to play important roles in endothelial cell invasion. We thus tested whether THL could inhibit the secretion of MMP-2 and uPA in endothelial cells. Zymography assays indicated that THL dose-dependently inhibited the secretion of MMP-2 (Fig 4C) and uPA (Fig 4D) in HMEC-1 cells. VEGF is known to be able to induce MMP-2 expression in endothelial cells [62]. We thus tested whether THL could inhibit VEGF-induced MMP-2 expression in endothelial cells. The data shown in Fig 4E indicate that this is indeed the case.

### THL inhibits tube formation by endothelial cells

**Figure 5:**
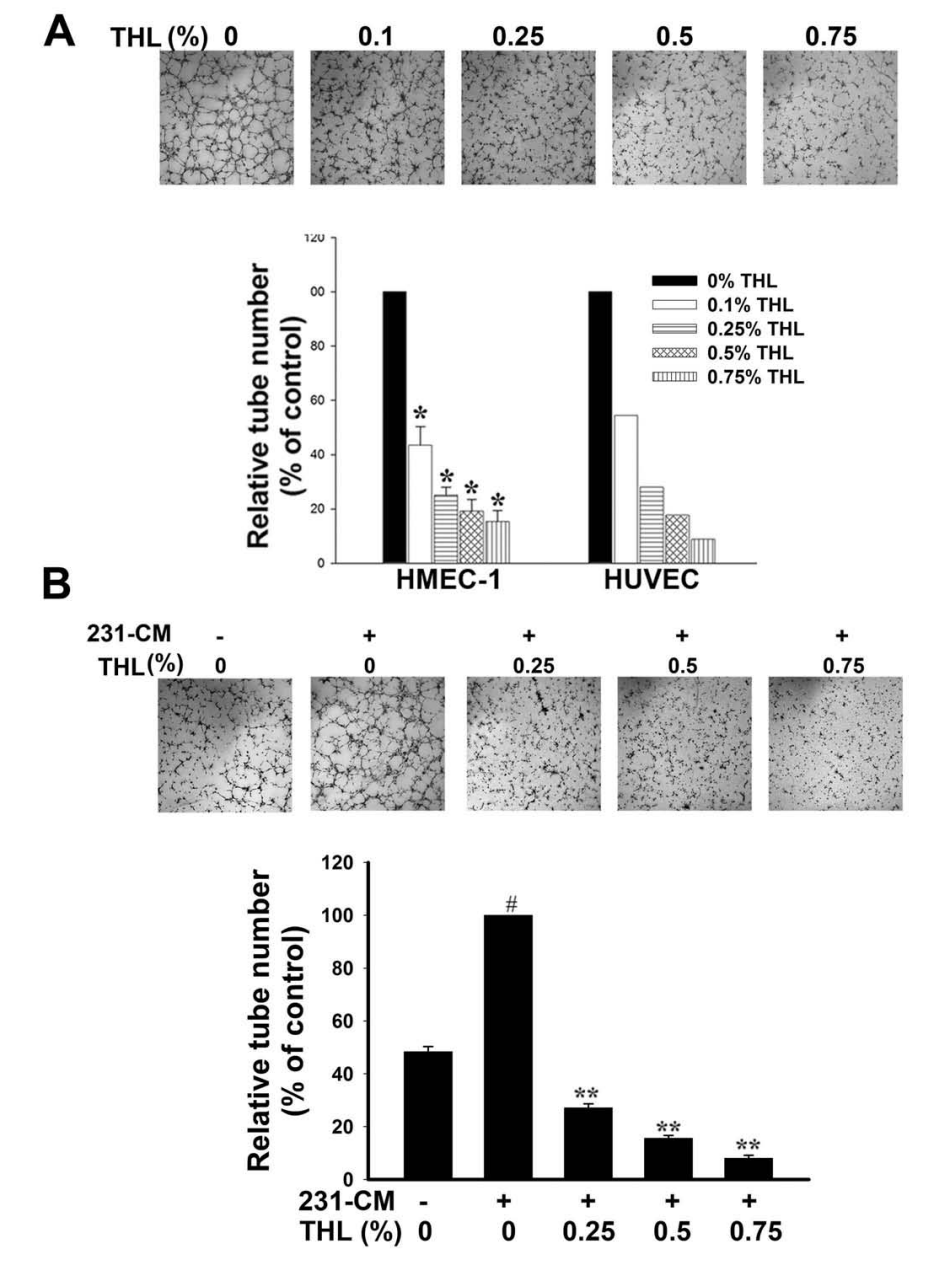
**THL inhibits the tube-formation ability of endothelial cells**. A) THL dose-dependently inhibits FBS-induced capillary-like tube formation by HMEC-1 and HUVEC cells. *Upper panel*, images of FBS-induced tube formation by HMEC-1 cells in the presence of various concentrations of THL. *Lower panel*, quantitation of FBS-induced tube formation by HMEC-1 and HUVEC cells in the presence of various concentrations of THL. The number of tubes was counted in six randomly selected microscopic fields (× 200 magnification). The relative tube number was obtained by comparing the number of tubes formed in the presence of THL with that formed in the absence of THL. Values represent means ± SD, n = 2. *P < 0.05 versus untreated control. B) THL dose-dependently inhibits 231-CM-induced capillary-like tube formation by HMEC-1 cells. *Upper panel*, images of tube formation by HMEC-1 cells in various conditions. *Lower panel*, quantitation of tube formation by HMEC-1 cells in various conditions. The number of tubes was counted at 5 h post-seeding in six randomly selected microscopic fields (× 200 magnification). The relative tube number was obtained by comparing the number of tubes formed in different conditions with that formed in the presence of 231-CM without THL. Values represent means ± SD, n = 2. ^#^*P *< 0.05 versus unstimulated control (in the absence of 231-CM and THL); **P < 0.001 versus 231-CM control (in the presence of 231-CM only).

In the latter stage of angiogenesis, endothelial cells will self-assemble into tubes to form new blood vessels. To investigate the effects of THL on neovascularization, HMEC-1 or HUVEC cells were cultured on the Matrigel-coated plates in the presence of the angiogenic stimulator (10% FBS) and various concentrations of THL for 5 h. As shown in Fig 5A upper panel, HMEC-1 cells formed the complete network structures in the presence of 10% FBS, indicating that FBS can induce tube formation of endothelial cells. In the presence of 10% FBS plus THL, tubular formation by HMEC-1 was significantly inhibited (Fig 5A, upper panel). The inhibitory effect of THL on tube formation induced by FBS was quantitated by counting the number of tubes. As shown in Fig 5A lower panel, the extent of tubular formation of HMEC-1 and HUVEC was reduced by THL in a dose-dependent manner. We also tested whether THL could inhibit 231-CM-induced tube formation by HMEC-1. 231-CM indeed could stimulate tube formation in HMEC-1 cells (Fig 5B). THL reduced the extent of tubular formation of HMEC-1 to levels even lower than that in the absence of 231-CM and this inhibition on tube formation was dependent on THL concentration (Fig 5B).

### THL inhibits angiogenesis in the Matrigel plug model

**Figure 6:**
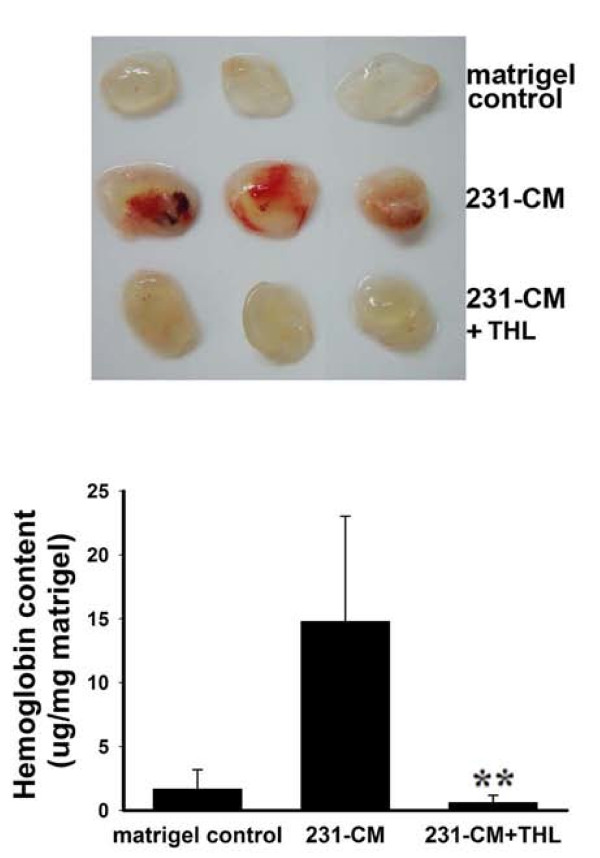
**Effect of THL on the 231-CM-induced angiogenesis in Matrigel plugs in NOD-SCID mice**. Upper panel, Representative pictures of control (without 231-CM), 231-CM-containing, 231-CM+THL-containing Matrigel plugs at Day 14 after implantation into mice. Lower panel, quantitation of hemoglobin level in the plugs. Values represent means ± SD, n = 5-7. **P < 0.001 versus 231-CM-containing plugs.

In order to investigate whether THL suppressed vascularization *in vivo*, the Matrigel plug assay was performed. In contrast to plugs without 231-CM (serves as an angiogenic stimulator), plugs loaded with 231-CM exhibited bright red color indicating that 231-CM can induce new blood vessel formation in Matrigel plugs (Fig 6). In the presence of THL, plugs showed light yellowish color indicating the absence of angiogenesis (Fig 6, upper panel). The extent of angiogenesis was quantified by measuring the hemoglobin content in the plugs. The amount of hemoglobin in the Matrigel plugs loaded with 231-CM plus THL was much lower than that in the plugs loaded with 231-CM alone (Fig 6, lower panel), suggesting that THL can inhibit neovascularization *in vivo*.

### THL inhibits the expression of HIF-1α and VEGF-A in MDA-MB-231 breast cancer cells

**Figure 7:**
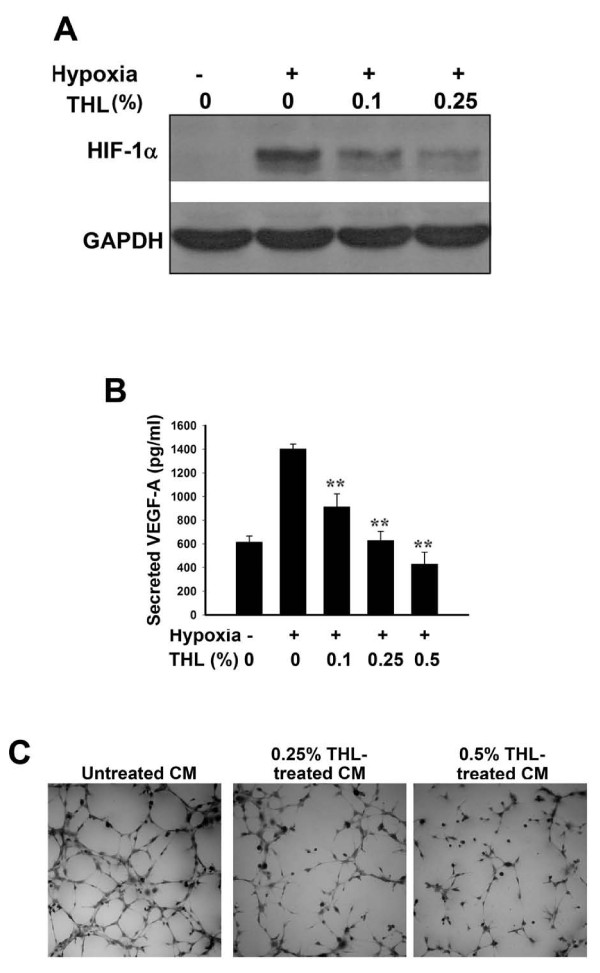
**THL inhibits hypoxia-induced HIF-1α and VEGF-A expression in MDA-MB-231 breast cancer cells**. MDA-MB-231 cells were cultured under normoxia or hypoxia (with 200 μM CoCl_2_, a hypoxia-mimetic agent, in the medium) in serum-free medium containing various concentrations of THL for 24 h. Protein level of HIF-1α in cell lysates was analyzed by Western blot A), and VEGF-A secreted into the conditioned medium was analyzed by ELISA B). For the secreted VEGF-A, values represent means ± SD, n = 4. **P < 0.001 versus untreated cells under hypoxia. The conditioned media from untreated and THL-treated cells under hypoxia were tested for their effect on HMEC-1 tube formation C).

In rapidly growing tumors, local hypoxic conditions induce the expression of transcription factor HIF-1α, which in turn activates the expression of VEGF in tumor cells. The VEGF secreted by tumor cells then binds to and activates the receptor on the surface of endothelial cells, leading to endothelial cell proliferation, migration, invasion, and eventually capillary tube formation. To suppress new blood vessel formation in tumors, it is important to inhibit the expression of HIF-1α and VEGF in tumor cells. As shown in Fig 7A, THL dose-dependently inhibited the hypoxia-induced HIF-1α expression in MDA-MB-231 cancer cells. As a result, the amount of VEGF-A secreted into the medium by MDA-MB-231 cells was also reduced by THL in a dose-dependent manner (Fig 7B). This inhibition of HIF-1α and VEGF-A expression is not due to cytotoxic effect of THL, because under similar hypoxic condition, THL treatment did not affect the viability of MDA-MB-231 cells (see Additional file 4: Supplemental figure S4). The conditioned media from the THL-treated and untreated MDA-MB-231 cells were tested for their potency to induce HMEC-1 tube formation. As shown in Fig 7C, the conditioned medium from THL-treated MDA-MB-231 cells had much lower potency to induce HMEC-1 tube formation than the control conditioned medium.

### THL inhibits the growth of human MDA-MB-231 breast cancer xenografts in SCID mice

**Figure 8:**
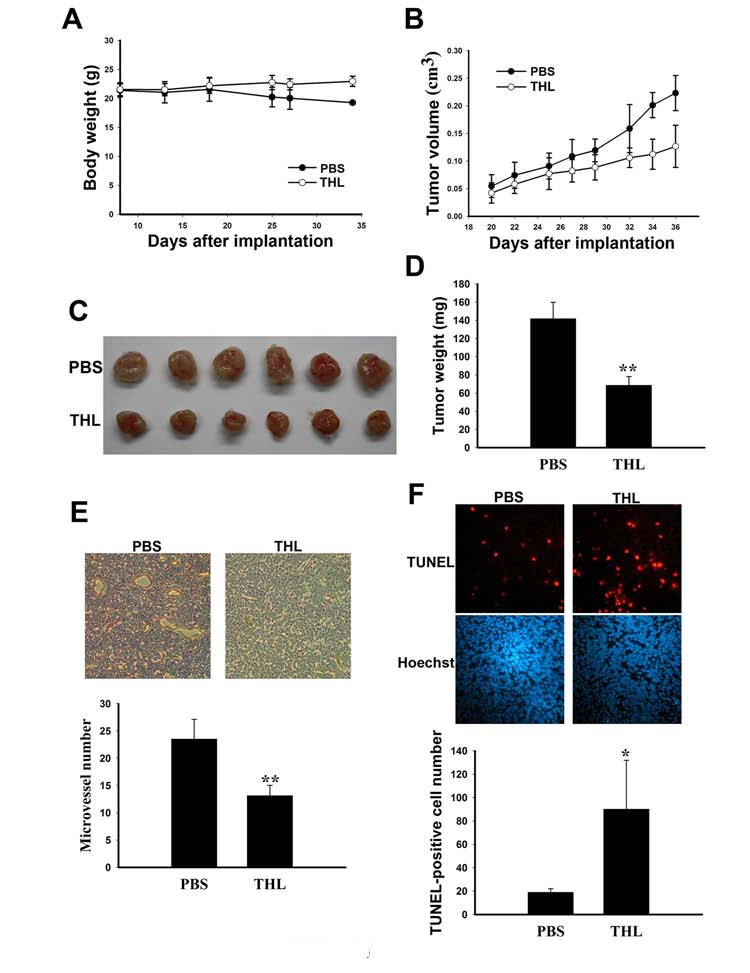
**THL inhibits the growth of human MDA-MB-231 breast cancer xenografts in SCID mice**. Effect of THL on the body weight A), tumor growth B), final tumor size C), and final tumor weight D) of NOD-SCID mice subcutaneously inoculated with human MDA-MB-231 breast cancer cells. For body weight, values represent means ± SD, n = 4 - 5 mice per group. For tumor volume data, values represent means ± SD, n = 8 - 10 tumors per group. For tumor weight data, values represent means ± SD, n = 6 tumors per group. **P < 0.001 versus PBS-treated mice. E) THL inhibits the angiogenesis of MDA-MB-231 tumors grown in NOD-SCID mice. Upper panel, images of CD31-positive stained microvessels in tumor sections of PBS- and THL-treated mice. Lower panel, quantitation of microvessels in tumor sections of PBS- and THL-treated mice. For each section, 4-6 randomly chosen fields were counted. Values represent means ± SD, n = 6 tumors per group. **P < 0.001 versus PBS-treated mice. F) THL induces the apoptosis of MDA-MB-231 tumors grown in NOD-SCID mice. Upper panel, images of TUNEL and Hoechst 33258 staining of tumor sections from PBS- and THL-treated mice. Lower panel, quantitation of TUNEL-positive cells in tumor sections of PBS- and THL-treated mice. For each section, 4-6 randomly chosen fields were counted. Values represent means ± SD, n = 6 tumors per group. *P < 0.05 versus PBS-treated mice.

The preceding data promoted us to assess the anti-tumor activity of THL *in vivo*. Female *NOD-SCID *mice were subcutaneously injected with 3 × 10^6 ^MDA-MB-231 breast cancer cells. One week later, mice were randomly divided into two groups: one group was intraperitoneally administered with THL and another group received PBS. Three weeks after tumor cell implantation, while the PBS-treated mice started to lose their body weight gradually, the THL-treated mice did not (Fig 8A). The PBS-treated mice appeared sick with bristly hair, in sharp contrast to THL-treated mice which looked much healthier with smooth and shiny hair. We also found that the growth of the tumors was slower in THL-treated mice than in PBS-treated mice (Fig 8B). The tumors were removed, photographed, and weighed at Day 36 after tumor cell implantation. As shown in Fig 8C and 8D, the tumors from THL-treated mice had much smaller size and weight than those from PBS-treated mice. The IHC analysis on tumor sections showed that tumors from THL-treated mice contained fewer CD31-positive stained microvessels than those from PBS-treated mice, consistent with the notion that THL can suppress tumor angiogenesis (Fig 8E). The TUNEL assay on tumor sections revealed that tumors from THL-treated mice contained much more apoptotic cells than those from PBS-treated mice (Fig 8F). Together, these data indicate that THL can induce tumor apoptosis and suppress tumor angiogenesis and growth in *SCID *mice. Since *SCID *mice are immunodeficient, the induction of apoptosis and suppression of tumor growth by THL does not require host immune function and most likely results from the anti-angiogenic or direct killing effect of THL.

## Discussion

Advanced cancer is a multifactorial disease that accumulates many genetic and epigenetic alterations affecting multiple distinct regulatory circuits within cells. To treat advanced cancer, there is a growing belief that combination therapy using multiple drugs targeting various cellular pathways would yield better outcomes than monotherapies. In this respect, herbal cocktail which contains various phytochemicals targeting multiple dys-regulated pathways in cancer cells may provide an alternative/complementary way to treat cancers. In this study, we demonstrated that the Chinese herbal cocktail THL not only could inhibit the *in vitro *migration and invasion ability of various cancer cells but also could inhibit the metastasis of colon cancer cells to lung in mice. Angiogenesis plays a critical role in the growth and metastasis of tumors. Our data indicate that THL not only could inhibit the migration, invasion, and tube formation ability of endothelial cells but also could inhibit cancer cells to secrete the pro-angiogenic factor VEGF-A. The *in vivo *anti-angiogenic activity of THL was also demonstrated in the Matrigel plug model and tumor xenograft in *NOD-SCID *mice. Together, these data indicate that THL is a potential cancer therapeutic agent and merits further evaluation for preventive and therapeutic application to human cancers.

The formation of distant metastasis is the main cause of morbidity and mortality in patients with cancer. In recent years, much effort has been taken to develop drugs that can inhibit metastasis. However, till now promising anti-metastatic agents are still lacking [5]. In this study, we demonstrated that THL could inhibit the *in vitro *migration and invasion ability of breast, lung, prostate, and colon cancer cells (Fig 1) and suppress the pulmonary metastasis of colon cancer cells in mice (Fig 3). Our data indicate that several activities of THL may account for its inhibitory effect on cancer metastasis. Firstly, THL could inhibit, in cancer cells, the expression and secretion of MMP-2, MMP-9, and uPA (Fig 2A, C), which involve in degradation of extracellular matrix and play important roles in cancer cell migration and invasion [5,6,63]. Secondly, THL could inhibit the activity of MMP-2 directly (Fig 2B). Thirdly, THL could inhibit, in cancer cells, the activity of ERK1/2, key molecules of the ERK signaling pathway that has been shown to promote tumor invasion and metastasis [61,64] (Fig 2D). Fourthly, THL could inhibit, in cancer cells, the expression of HIF-1α (Fig 7A), a transcription factor that promotes metastasis by regulating the expression of metastasis-related genes [10]. Together, these data suggest that THL has multiple anti-metastatic activities and has the potential to be developed into an anti-metastatic agent. This argument is further supported by the observation that THL could inhibit angiogenesis, a critical process for cancer cells to spread to other organs.

We demonstrated that THL could suppress tumor angiogenesis in immunodeficient *NOD-SCID *mice (Fig 8E). We also demonstrated that THL could inhibit *in vivo *neovascularization in Matrigel plugs loaded with cancer cell-derived conditioned medium (231-CM) (Fig 6). At least two activities of THL may contribute to its anti-angiogenic effect in tumors. Firstly, THL could directly inhibit the migration, invasion and tube formation of endothelial cells (Fig 4 and 5). Secondly, THL could inhibit the secretion of pro-angiogenic factor by cancer cells (Fig 7). With regard to the direct inhibitory effect on endothelial cells, we have found that THL could inhibit the expression of MMP-2 (Fig 4C) and uPA (Fig 4D) in endothelial cells. MMPs and uPA are known to play a critical role in endothelial cell migration and invasion [8,63]. The inhibition of MMPs and uPA expression in endothelial cells can lead to suppression of endothelial cell migration and invasion. With regard to the effect of THL on cancer cells, we have found that THL could inhibit the secretion of VEGF-A by cancer cells (Fig 7B). At least two mechanisms may account for THL inhibition of expression of VEGF-A in cancer cells. Firstly, we found that THL could inhibit the hypoxia-induced expression of HIF-1α which is a transcriptional activator of the *vegf-A *gene [10], in cancer cells (Fig 7A). Secondly, we found that THL could inhibit, in cancer cells, the activity of ERK1/2 (Fig 2D), which phosphorylates the transcription factor Sp1 and causes the recruitment of Sp1 to the *vegf-A *promoter [65]. Our finding that THL can inhibit cancer cells to express HIF-1α and to secrete VEGF-A is important in terms of suppression of tumor angiogenesis. To induce neovascularization in tumors, tumor cells must secrete pro-angiogenic factors, such as VEGF-A, which attracts and guides sprouting neovessels into oxygen-depleted regions of the tumor mass. The blocking of HIF-1α and VEGF-A expression in tumor cells can thus lead to inhibition of neovessel formation in tumors.

This study has identified multiple biological pathways as potential targets of the anti-tumor activities of the THL formula. It will be of interest to identify the active chemical compounds in the formula that target these pathways. We have started to fractionate THL by ethyl acetate partition and silica gel column chromatography. Our preliminary results indicated that several fractions of THL could inhibit the migration/invasion ability of H1299 cancer cells. Among them, fraction 4 had the strongest activity in inhibiting H1299 migration/invasion ability (data not shown). Experiments using high-performance liquid chromatography to isolate active components of fraction 4 are in progress. Conceptually, it will be interesting and important to determine whether the anti-metastatic and anti-tumor activities of THL can be reconstituted by a few active chemical compounds identified from THL.

## Conclusions

Chinese herbal cocktails designed to maximize the synergistic and minimize the antagonistic interactions among various phytochemicals present in different herbs may have therapeutic efficacy against multifactorial diseases such as cancer. In this study, we demonstrated that the Chinese herbal cocktail THL could inhibit cancer metastasis and angiogenesis by targeting multiple biological and pathological processes in cancer cells. We also demonstrated that THL, which was delivered one week after tumor implantation, could exert anti-tumor effects but had no adverse effect on body weight in immuno-compromised mice, implicating that THL has potential to be used as a therapeutic agent for established tumors. Moreover, our preliminary results also indicated that oral delivery of THL could inhibit tumor growth and induce anti-tumor immunity in immuno-competent mice (J-L Du and W-B Wang, data not shown). Together these data suggest that THL is a promising cancer therapeutic agent and merits further investigation.

## Competing interests

The authors declare that they have no competing interests. All authors are employees of College of Medicine, National Taiwan University and this study was funded by Ching-Hsing Medical Foundation, a nonprofit organization in Taiwan.

## Authors' contributions

JSC designed and helped perform the *in vitro *and *in vivo *experiments and drafted the original manuscript. JLD performed the *in vitro *and *in vivo experiments and helped draft the original manuscript*. WBH, helped perform the tumor xenograft *experiments*. AS and CPC assisted in the study design and interpretation of the data. WBW supervised and coordinated the study and finalized the manuscript. All authors read and approved the manuscript.

## Pre-publication history

The pre-publication history for this paper can be accessed here:

http://www.biomedcentral.com/1471-2407/10/175/prepub

## Supplementary Material

Additional file 1 **Supplementary figure S1. Effect of THL on the viability of MDA-MB-231, H1299, PC-3 and CT-26 cancer cells during 6-h treatment period**. Cancer cells (5000 cells/well) were seeded in 96-well plates overnight and fed with fresh medium containing various concentrations of THL for 6 h. The cell viability was measured by 3-(4,5-dimethyl-thiazol-2-yl) 2,5-diphenyl tetrazolium bromide (MTT) assay. The MTT assay was performed as follows. After incubation in THL-containing medium, cells were incubated with 0.4 mg/ml MTT (Sigma, St. Louis, MO) at 37°C for 3 h. Cells were then dissolved in DMSO at 37°C for 5 min and the spectrophotometric absorbance of the samples was determined by using ELISA reader (Biotek, Winooski, VT) at 550 nm. Values represent means ± SD, n = 4. *P < 0.05 versus untreated control. Click here for file

Additional file 2 **Supplementary figure S2. THL inhibits the transcription of the MMP-2 and MMP-9 genes**. MDA-MB-231 breast cancer cells were either untreated or treated with 0.5% THL for 24 h. The level of MMP-2 and MMP-9 mRNA expressed in the cells was then quantitated by real-time RT-PCR using primer pairs specific for MMP-2 and MMP-9. The level of mRNA expressed in the untreated cells was set as 1. Values represent means ± SD, n = 2. *P < 0.05 versus untreated control. Click here for file

Additional file 3 **Supplementary figure S3. Effect of THL on the viability of HMEC-1 and HUVEC endothelial cells during 6-h treatment period**. Cells (5000 cells/well) were seeded in 96-well plates overnight and fed with fresh medium containing various concentrations of THL for 6 h. The cell viability was measured by MTT assay. Values represent means ± SD, n = 4. Click here for file

Additional file 4 **Supplemental figure S4. Effect of THL on the viability of MDA-MB-231 cancer cells under hypoxic condition**. Cells (5000 cells/well) were seeded in 96-well plates overnight and then incubated under normoxia or hypoxia in serum-free medium containing various concentrations of THL for 24 h. The cell viability was measured by MTT assay. Values represent means ± SD, n = 6. Click here for file
