# Supplementary figures and images for: Inhibition of metastasis, angiogenesis, and tumor growth by Chinese herbal cocktail Tien-Hsien Liquid
Source: BMC Cancer. 2010 Apr 30;10:175. doi: 10.1186/1471-2407-10-175 (PMC2880989; doi:10.1186/1471-2407-10-175)

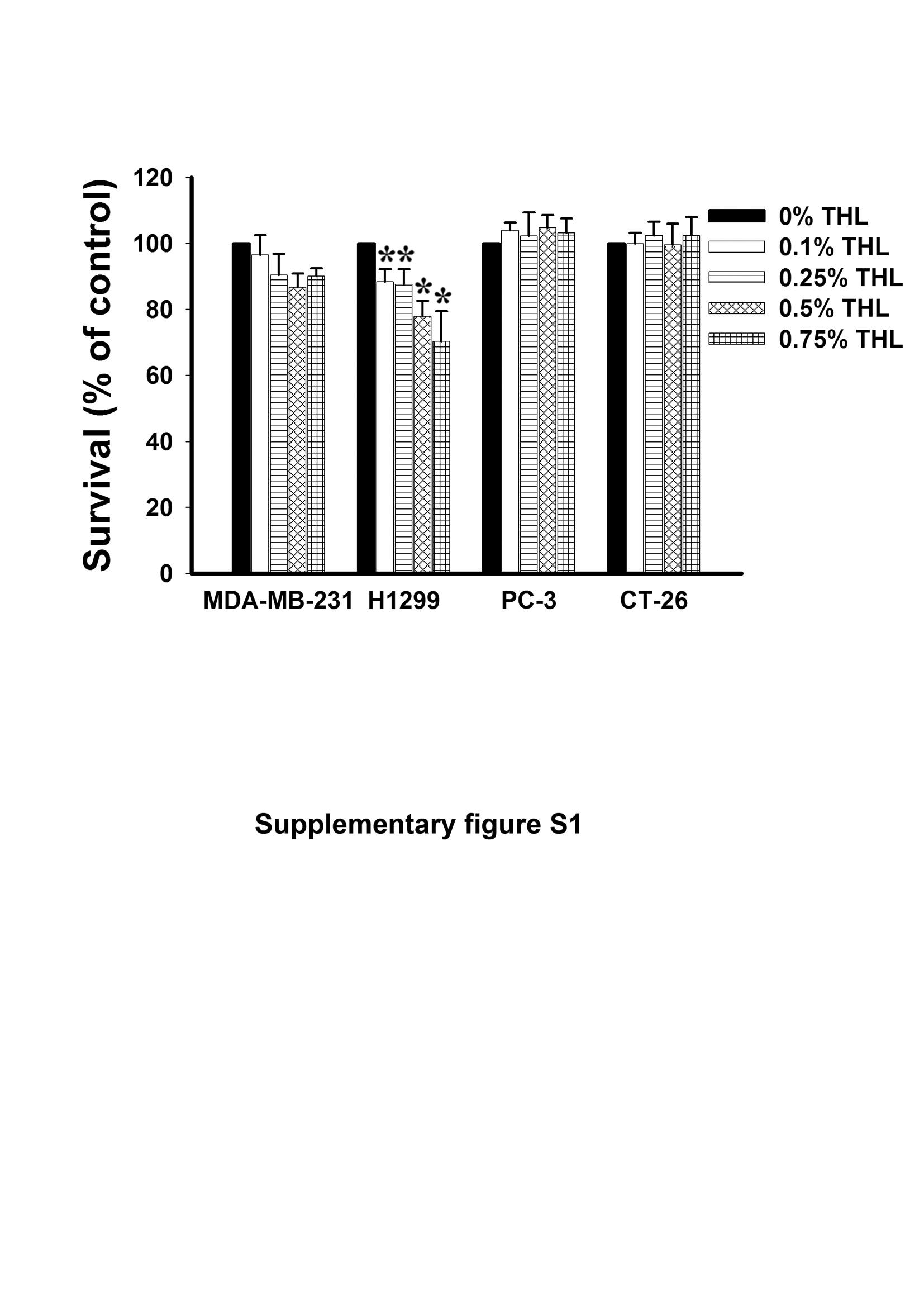

Supplement: Additional file 1 — Supplementary figure S1. Effect of THL on the viability of MDA-MB-231, H1299, PC-3 and CT-26 cancer cells during 6-h treatment period. Cancer cells (5000 cells/well) were seeded in 96-well plates overnight and fed with fresh medium containing various concentrations of THL for 6 h. The cell viability was measured by 3-(4,5-dimethyl-thiazol-2-yl) 2,5-diphenyl tetrazolium bromide (MTT) assay. The MTT assay was performed as follows. After incubation in THL-containing medium, cells were incubated with 0.4 mg/ml MTT (Sigma, St. Louis, MO) at 37°C for 3 h. Cells were then dissolved in DMSO at 37°C for 5 min and the spectrophotometric absorbance of the samples was determined by using ELISA reader (Biotek, Winooski, VT) at 550 nm. Values represent means ± SD, n = 4. *P < 0.05 versus untreated control. [file 1471-2407-10-175-S1.TIFF]

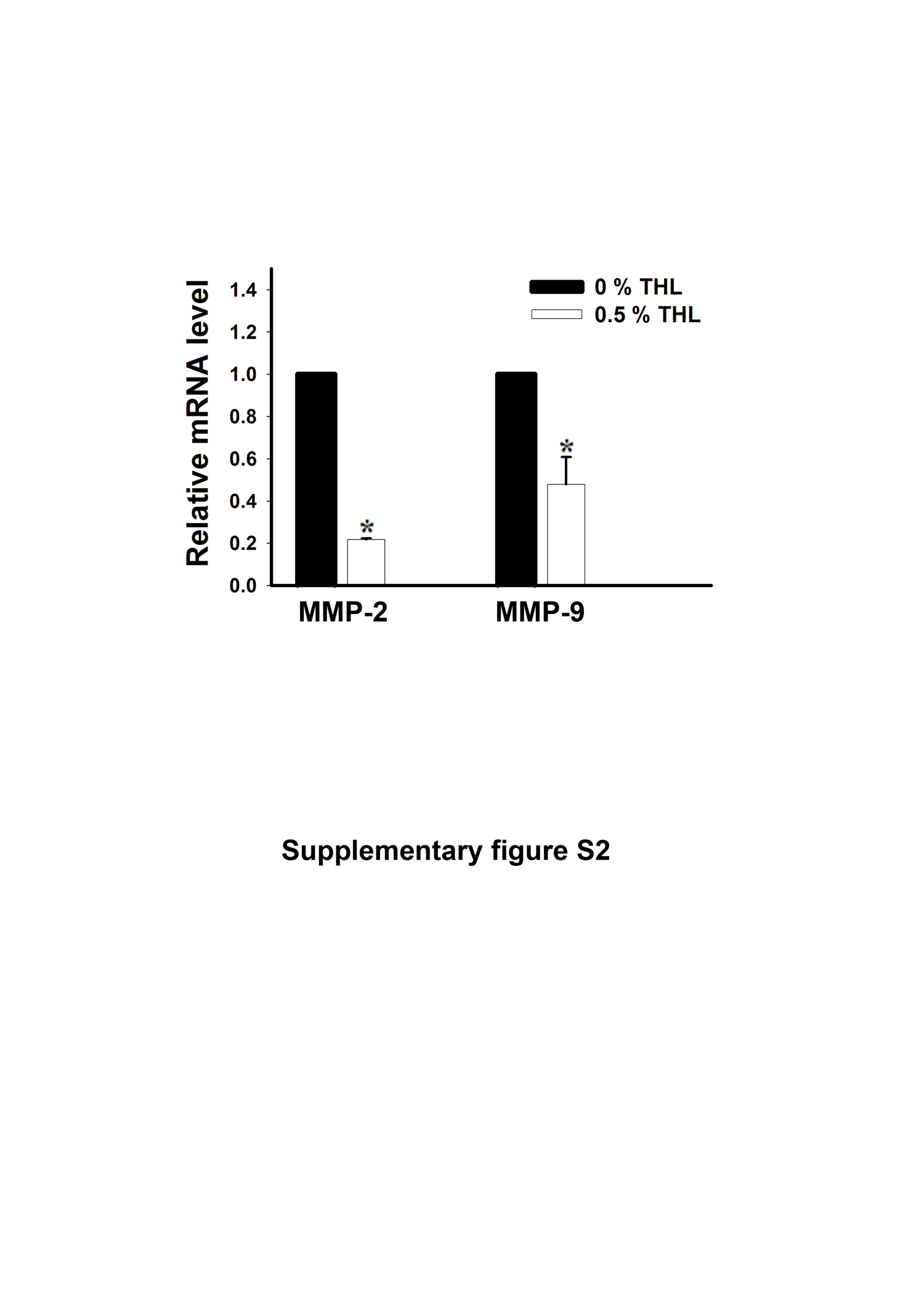

Supplement: Additional file 2 — Supplementary figure S2. THL inhibits the transcription of the MMP-2 and MMP-9 genes. MDA-MB-231 breast cancer cells were either untreated or treated with 0.5% THL for 24 h. The level of MMP-2 and MMP-9 mRNA expressed in the cells was then quantitated by real-time RT-PCR using primer pairs specific for MMP-2 and MMP-9. The level of mRNA expressed in the untreated cells was set as 1. Values represent means ± SD, n = 2. *P < 0.05 versus untreated control. [file 1471-2407-10-175-S2.TIFF]

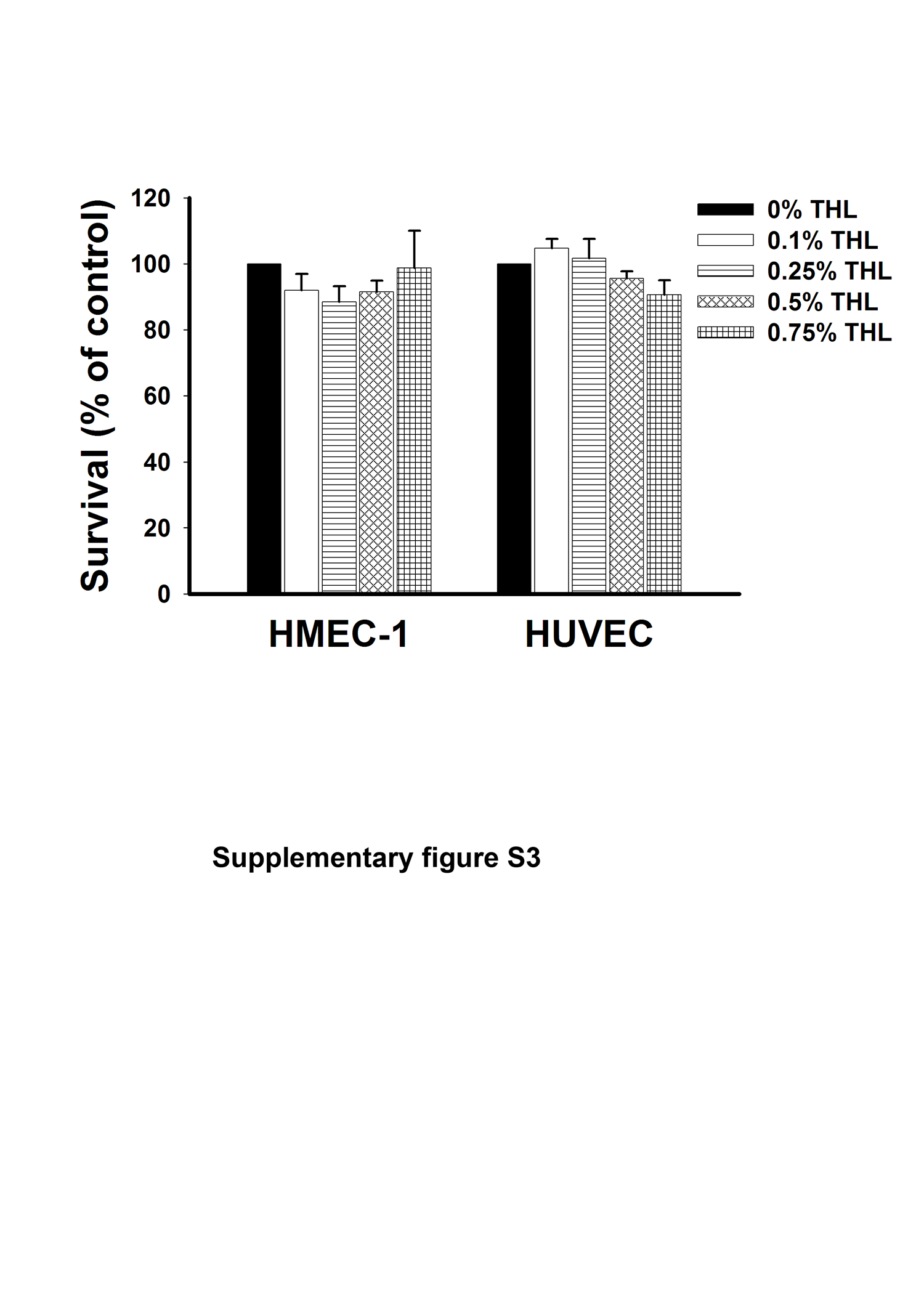

Supplement: Additional file 3 — Supplementary figure S3. Effect of THL on the viability of HMEC-1 and HUVEC endothelial cells during 6-h treatment period. Cells (5000 cells/well) were seeded in 96-well plates overnight and fed with fresh medium containing various concentrations of THL for 6 h. The cell viability was measured by MTT assay. Values represent means ± SD, n = 4. [file 1471-2407-10-175-S3.TIFF]

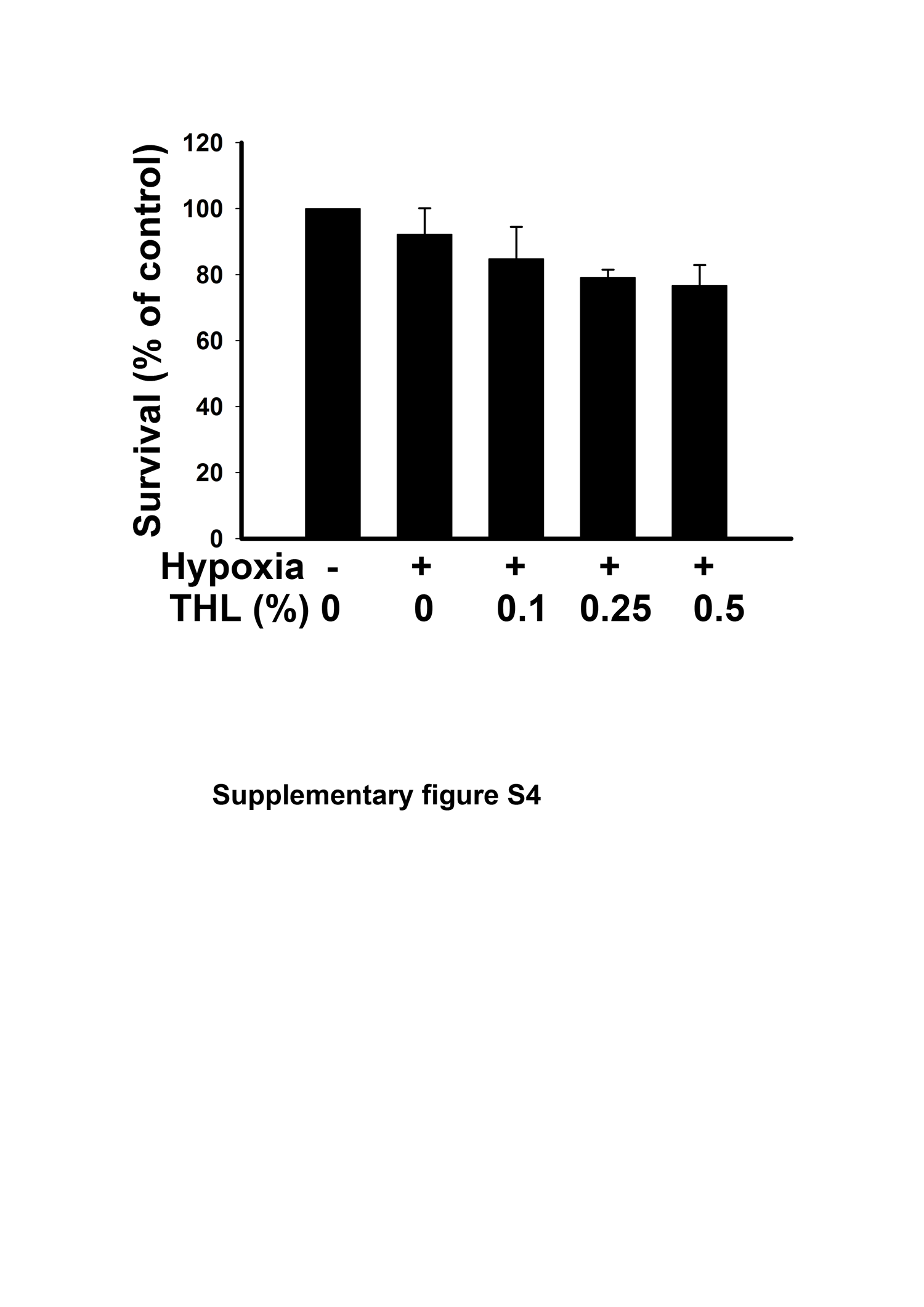

Supplement: Additional file 4 — Supplemental figure S4. Effect of THL on the viability of MDA-MB-231 cancer cells under hypoxic condition. Cells (5000 cells/well) were seeded in 96-well plates overnight and then incubated under normoxia or hypoxia in serum-free medium containing various concentrations of THL for 24 h. The cell viability was measured by MTT assay. Values represent means ± SD, n = 6. [file 1471-2407-10-175-S4.TIFF]
